# Non-invasive assessment of estimated glomerular filtration rate using lens autofluorescence in a population without chronic kidney disease

**DOI:** 10.3389/fmed.2026.1821552

**Published:** 2026-09-30

**Authors:** Dan Cao, Shaoyun Li, Yuefei Li, Jian Shao, Li Qu, Kaixin Zhou, Yang Cao

**Affiliations:** 1 Renji Hospital, School of Medicine, Chongqing University, Chongqing, China; 2 The Fifth People’s Hospital of Chongqing, Chongqing, China; 3 Guangzhou Laboratory, Guangzhou, Guangdong, China

**Keywords:** advanced glycation end products, estimated glomerular filtration rate, lens autofluorescence, chronic kidney disease, non-invasive assessment

## Abstract

**Purpose:**

The estimated glomerular filtration rate (eGFR) is a vital indicator for monitoring renal health. Limitations caused by invasive serum-based measurement often delay the detection of early-stage renal decline. Advanced glycation end products (AGEs) contribute significantly to renal pathogenesis. This study specifically targets the “pre-clinical” window of renal decline. We investigate whether lens autofluorescence based AGE (LAF-AGE) measurement can serve as a non-invasive early warning indicator for eGFR in an asymptomatic, non chronic kidney disease (CKD) control population, facilitating the possibilities of detection of individuals at risk of future CKD before clinical manifestations.

**Methods:**

From an initial pool of 1,937 participants, we excluded those with eGFR < 60 mL/min or albuminuria or microalbuminuria (*n* = 1,725). Following the removal of biochemical outliers using the interquartile range (IQR) method, 1,287 participants were included in the final analysis. LAF-AGEs were measured using a scanning confocal biomicroscope. Age-adjusted AGE levels (AALs) were derived based on population percentiles. Multivariate linear regression was used to evaluate associations while adjusting for confounders, including sex, BMI, blood glucose, and lipids.

**Results:**

LAF-AGEs showed a significant negative association with eGFR in the univariate model (*β* = −47.80, *p* < 0.001). This association remained significant after adjusting for confounders. Higher AALs were independently associated with lower eGFR (*p* = 0.002).

**Conclusion:**

LAF-AGEs is a valid, practical and non-invasive parameter for eGFR evaluation.

## Introduction

Chronic kidney disease (CKD) causes irreversible damage to the kidneys and is a major cause of preventable morbidity (1). The clinical standard for assessment is the estimated glomerular filtration rate (eGFR), which reflects the volume of blood filtered by the renal glomeruli per minute (2, 3). The progressive decline in eGFR indicates the loss of functional nephron units responsible for the clearance of metabolic waste products, maintaining mineral and bone homeostasis, and regulating systemic cardiovascular health.

eGFR is calculated using a formula that takes into account a person's age, sex, race, and serum creatinine level (CKD-EPI equation) (4). Invasive eGFR measurement has several limitations. Patients may experience discomfort or anxiety associated with blood sampling. The procedure is time-consuming, and variations exist in calculated eGFR values. Collectively, these drawbacks can hinder the early detection of CKD. It would be of value to investigate a non-invasive measure that can reliably reflect eGFR and be used for the early screening of CKD.

Advanced glycation end products (AGEs) are compounds formed when proteins or lipids undergo glycation, a non-enzymatic reaction with reducing sugars (5, 6). Lens crystallins, nerve myelin, and skin collagen are primary sites where AGEs accumulate and are irreversibly stored within long-lived proteins over a person's lifetime (7, 8). The accumulation of AGEs in the kidneys and their subsequent effects on inflammation, fibrosis, oxidative stress, and endothelial dysfunction contribute to the pathogenesis and progression of CKD (9, 10). These findings indicate that AGE production and its accumulation are implicated in CKD, serving as more than a simple biomarker for glucose homeostasis (11).

Lens autofluorescence (LAF) is a phenomenon occurring when specific substances within the lens of the eye emit light upon excitation by specific wavelengths. Measured via scanning confocal biomicroscopy, LAF provides a novel, rapid, and non-invasive method for quantifying the accumulation of AGEs (12). Unlike transient serum AGEs, lens crystallins are long-lived proteins that are not regulated by insulin. They irreversibly accumulate AGEs, making them superior indicators of long-term metabolic stress (13).

While studies have linked LAF-AGEs to eGFR in diabetic populations (12, 14), it remains unclear if this relationship persists in asymptomatic, non-CKD individuals. Unlike transient serum markers, lens crystallins are long-lived proteins that are not regulated by insulin; they act as a stable “metabolic memory” of lifetime oxidative stress. This study fills a gap by evaluating LAF technology as a non-invasive tool for early renal screening,which may be applied for the early detection of chronic kidney disease later.

## Methods

### Participant selection and data quality

A total of 1,937 adult participants from the Physical Examination Center of the Fifth People's Hospital of Chongqing between February and December 2019 during their annual physical check-ups were initially recruited. We collected routine demographic and biochemical data, including age, sex, body mass index (BMI), fasting glucose (FG), uric acid (UA), triglycerides (TG), total cholesterol (TC), low-density lipoprotein (LDL), and mean arterial pressure (MAP). Apart from sex and BMI, the other serum measures were obtained through a blood test. The eGFR was calculated using the Chronic Kidney Disease Epidemiology Collaboration (CKD-EPI) equation (4).

To focus on early screening in a non-CKD population, individuals with albuminuria, microalbuminuria or an eGFR < 60 mL/min/1.73 m^2^ were excluded, leaving 1,725 participants. To ensure statistical rigor and data quality, the IQR method was applied to nine biochemical indicators to remove extreme outliers for the indicators [eGFR, AGEs (LAF-AGEs), age, FG, TG, TC, MAP, and LDL]. The final analysis included 1,287 participants (Figure 1).

**Figure 1:**
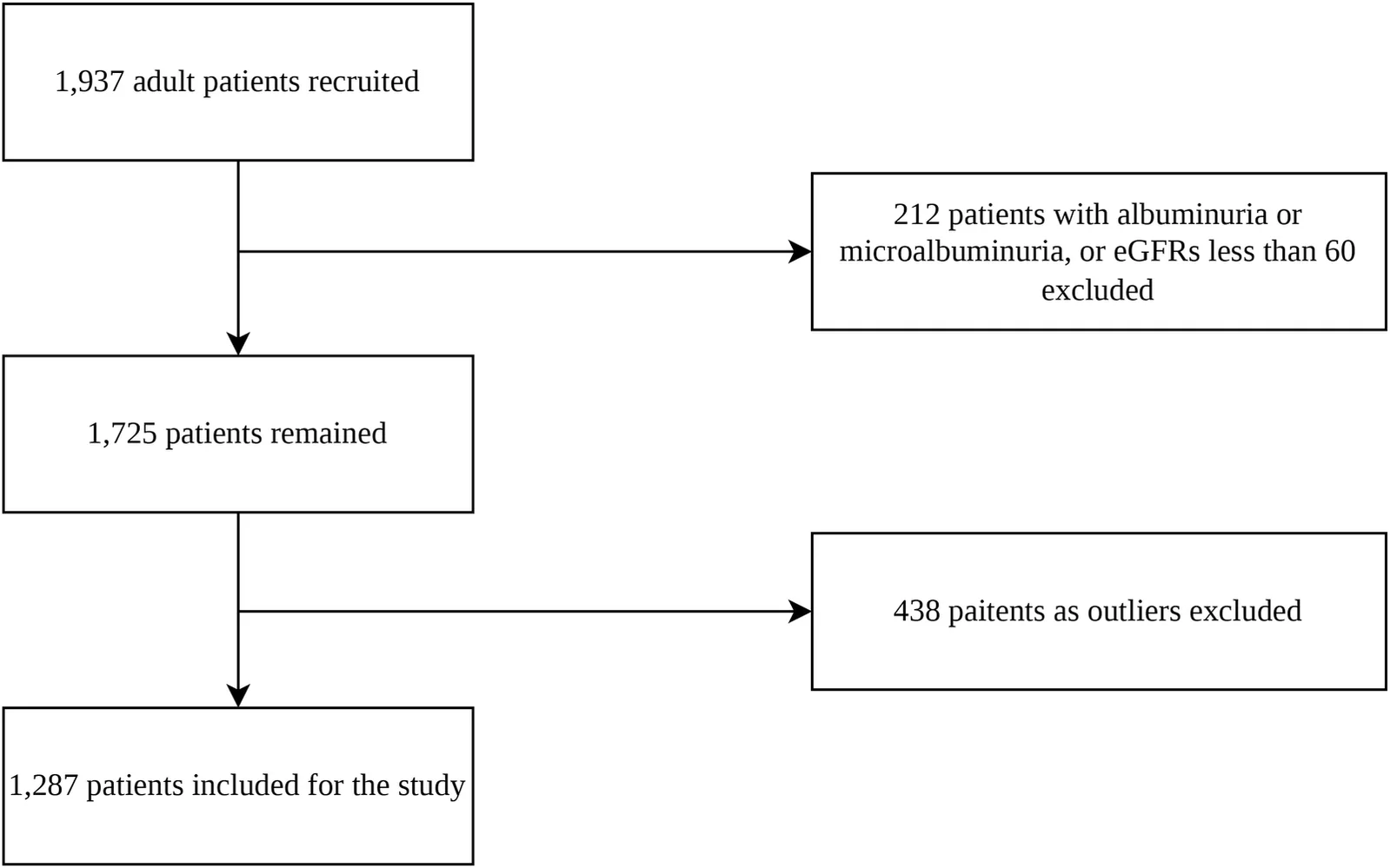
Flow diagram of the steps for study inclusion.

### Measurement of AGEs

Lens Autofluorescence Measurement: In this study, the AGEscan/ClearPath DS-120 Lens Fluorescence Biomicroscope was utilized to perform the measurement, which is completed in approximately 6 s. The measurement mechanism involves calculating the ratio of green fluorescence to scattered light, which serves as the raw LAF-AGE value (12). Importantly, this procedure requires no fasting or specific physiological preparation by the participant, making it a highly practical tool for early clinical screening.

Age-Adjusted AGE Levels (AALs): The raw LAF-AGE values were further adjusted by age and referenced against a normal population profile to derive four age-adjusted AGE levels (AALs) (12):

- Level 0 (Normal): below 50th percentile
- Level 1 (Low Risk): 50th–80th percentile
- Level 2 (Medium Risk): 80th–95th percentile
- Level 3 (High Risk): above 95th percentile

### Statistical analysis

Linear regression assumptions, including linearity and normality, were verified prior to analysis. To address potential multicollinearity, assessments were performed between lipid variables, especially TC and LDL. Continuous variables are expressed as mean ± standard deviation (SD), while categorical variables are reported as frequencies and percentages. Univariate linear regression analyses were conducted to evaluate the association between eGFR and both LAF-AGEs and AALs. The independent impact of LAF-AGEs on eGFR was further examined through multivariate linear regression, adjusting for potential confounders including sex, BMI, FG, UA, TG, TC, and LDL. Statistical significance was defined as a *p*-value <0.05. Data processing was performed using Python (version 3.8.5) and statistical analyses were conducted using R (version 4.0.3).

## Results

### Characteristics of the study population

Drawing on established data regarding the prevalence of CKD in China, metabolic-related risk factors such as hypertension, diabetes, dyslipidemia, and hyperuricemia were identified as significant contributors to renal function decline (12). Consequently, these variables were selected as confounders for the current study, as detailed in Table 1.

**Table 1:** Characteristics of the participants.

Characteristics	60 ≤ eGFR < 90 Number: 184	eGFR ≥ 90 Number: 1,103	*p* value
LAF-AGEs	0.22 ± 0.06	0.21 ± 0.06	0.007
Age	53.21 ± 10	44.81 ± 12	<0.001
Sex (%)			<0.001
Female	63 (34%)	505 (46%)	
Male	121 (66%)	598 (54%)	
BMI	24.24 ± 2.86	23.75 ± 3.33	0.03
FG	5.35 ± 0.52	5.23 ± 0.47	0.007
UA	383.92 ± 97.78	349.00 ± 86.22	<0.001
TG	1.61 ± 0.68	1.49 ± 0.71	0.02
TC	4.97 ± 0.86	4.80 ± 0.81	0.014
LDL	2.96 ± 0.70	2.82 ± 0.65	0.01
MAP	92.09 ± 12.06	89.06 ± 11.69	0.002

LAF-AGEs, lens autofluorescence; BMI, based AGEs body mass index; FG, fasting glucose; UA, uric acid; TG, triglycerides; TC, total cholesterol; LDL, low-density lipoprotein; MAP, mean arterial pressure.

Initially, participants with albuminuria, microalbuminuria, or an eGFR below 60 mL/min/1.73 m^2^ were excluded, leaving 1,725 individuals. Following the subsequent removal of statistical outliers (identified using the 1.5 × IQR method across nine biochemical indicators), the final analysis included 1,287 participants, whose characteristics are summarized in Table 1. The cohort was divided into two groups: a low eGFR group (60 ≤ eGFR < 90 mL/min/1.73 m^2^) and a high eGFR group (eGFR ≥ 90 mL/min/1.73 m^2^). Participants in the high eGFR group were generally younger, exhibited lower body mass indices (BMI), and maintained lower mean arterial pressure (MAP) compared to those in the low eGFR group.

### Association of LAF-AGEs and selected confounders with eGFR

LAF-AGEs demonstrated a significant negative association with eGFR (*β* = −47.80, *p* < 0.001) in the univariate linear regression model, as shown in Table 2. Univariate analyses of the other selected confounders also revealed significant associations with eGFR, with all *p*-values recorded below 0.001. These determinants were subsequently included in a multivariate linear regression analysis to evaluate their independent effects. In the fully adjusted multivariate model, LAF-AGEs remained a statistically significant independent factor (*β* = −43.37, *p* < 0.001) despite adjustments for FG and other metabolic confounders. This association demonstrated high statistical robustness, furthermore maintaining significance after accounting for the False Discovery Rate (FDR) with Benjamini–Hochberg (BH) adjustment across all included metabolic and demographic confounders. This confirms that the observed negative association with eGFR is independent of diabetic status within the control population. Conversely, BMI, TG and LDL were no longer significant (*p* = 0.33, *p* = 0.55 and *q* = 0.0514, respectively), indicating that their influence on eGFR was attenuated after accounting for other metabolic variables.

**Table 2:** Univariant model and multivariant model for confounders and eGFR.

Confounders	Univariant model		Multivariant model		
	*β*	*p* value	*β*	*p* value	*q* value (BH-adjusted)
LAF-AGEs	−47.80	<0.001	−43.37	<0.001	<0.01
Sex	−4.52	<0.001	−2.81	0.001	0.002
BMI	−0.51	<0.001	−0.12	0.33	0.371
FG	−5.39	<0.001	−2.44	0.002	0.0036
UA	−0.03	<0.001	−0.01	0.01	0.015
TG	−2.30	<0.001	0.35	0.55	0.55
TC	−2.75	<0.001	−3.89	<0.001	<0.001
LDL	−3.31	<0.001	2.99	0.04	0.0514
MAP	−2.23	<0.001	−0.11	<0.001	<0.001

LAF-AGEs, lens autofluorescence based AGEs; AAL, adjusted AGE levels; BMI, body mass index; FG, fasting glucose; UA, uric acid; TG, triglycerides; TC, total cholesterol; LDL, low-density lipoprotein; MAP, mean arterial pressure.

The distribution of eGFR and raw LAF-AGEs is illustrated in Figure 2A, where LAF-AGEs demonstrate a significant negative association with eGFR (*p* < 0.001). Further associations between LAF-AGEs and eGFR, following adjustments for specific individual confounders, are presented in Figures 2B–H. These visualizations confirm that the significant negative association between LAF-AGEs and eGFR remains robust even after accounting for each independent confounder.

**Figure 2:**
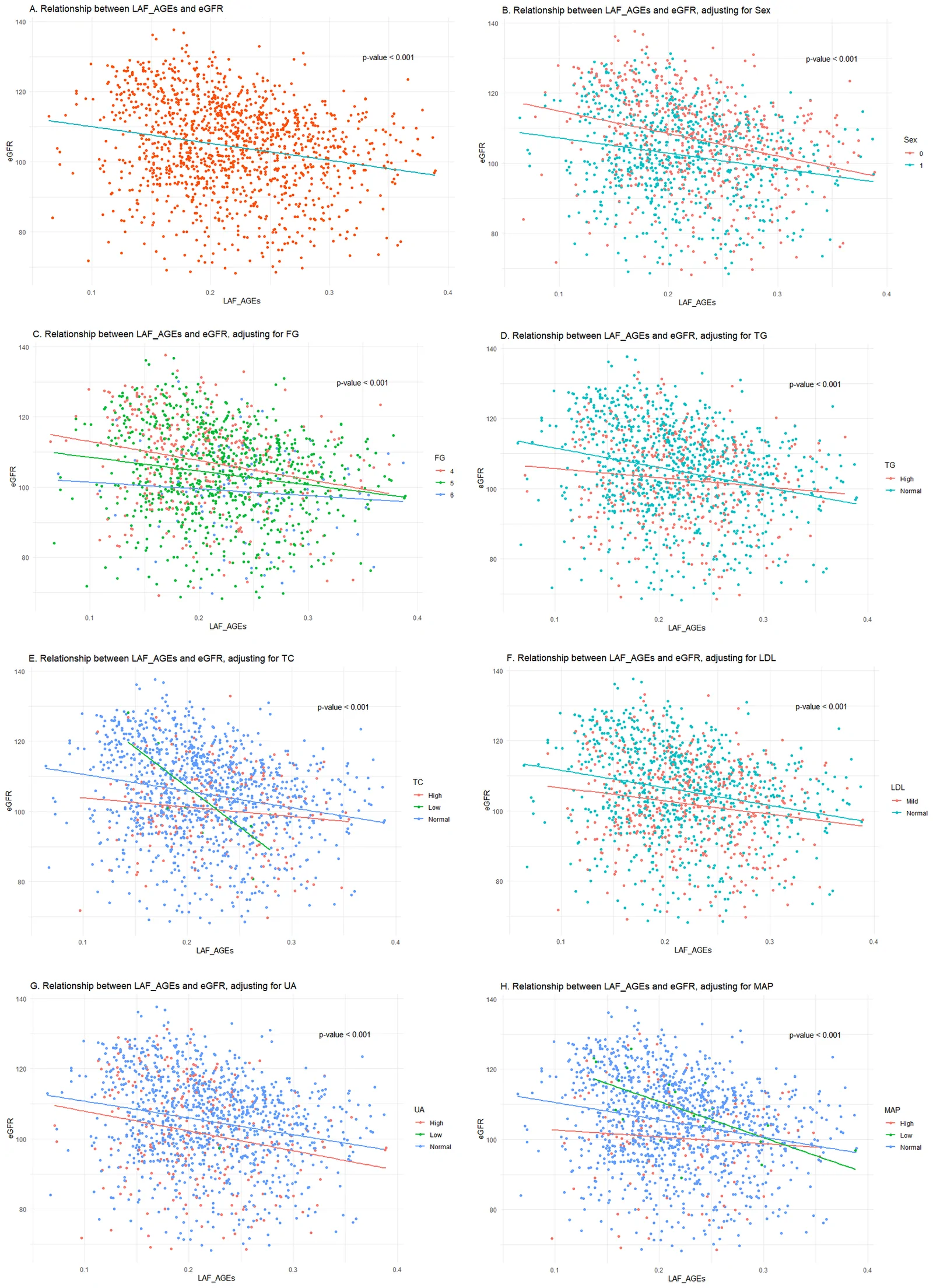
Correlation analysis between LAF-AGEs and eGFR. **(A)** Association between LAF-AGEs and eGFR; **(B)** association between LAF-AGEs and eGFR, adjusting for sex; **(C)** association between LAF-AGEs and eGFR, adjusting for FG; **(D)** association between LAF-AGEs and eGFR, adjusting for TG; **(E)** association between LAF-AGEs and eGFR, adjusting for TC; **(F)** association between LAF-AGEs and eGFR, adjusting for LDL; **(G)** association between LAF-AGEs and eGFR, adjusting for UA; **(H)** association between LAF-AGEs and eGFR, adjusting for MAP.

### Association of AAL with eGFR

Further analysis was performed to evaluate the relationship between AALs and eGFR. Univariate linear regression revealed a significant negative association (*β* = −1.49, *p* < 0.001). This association remained significant in the multivariate regression model (Table 3), which was adjusted for established clinical confounders. The results of this model demonstrated that AAL (*β* = −1.02, *p* = 0.002), alongside sex, FG, UA, TC, LDL, and MAP, were all significantly associated with eGFR. Conversely, BMI and TG did not reach statistical significance in this model.

**Table 3:** Linear regression analysis result.

Confounders	*β*	*p* value	*q* value (BH-adjusted)
AAL	−1.02	0.002	0.045
Sex	−2.55	0.004	0.072
BMI	−0.11	0.40	0.45
FG	−3.21	<0.001	<0.001
UA	−0.01	0.027	0.039
TG	0.21	0.72	0.72
TC	−4.26	<0.001	<0.001
LDL	3.24	0.031	0.039
MAP	−0.16	<0.001	<0.001

LAF-AGEs, lens autofluorescence based AGEs; AAL, adjusted AGE levels; BMI, body mass index; FG, fasting glucose; UA, uric acid; TG, triglycerides; TC, total cholesterol; LDL, low-density lipoprotein; MAP, mean arterial pressure.

## Discussion

It is recognized that eGFR is a crucial indicator used for the identification and monitoring of kidney disease as a decrease in eGFR serves as an early sign of renal damage or dysfunction (15). Currently, the glomerular filtration rate (GFR) is determined primarily through two methods. The first involves calculating eGFR using a specific formula, such as the CKD-EPI equation, which incorporates factors including age, sex, ethnicity, and serum creatinine levels (4). However, this method is invasive, as it requires a blood test that can cause patient anxiety. The second approach relies on isotope detection to accurately assess individual renal function (16); nonetheless, this method involves radiation exposure, which may pose potential risk to the patient. In this study, we provided evidence of an alternative non-invasive biomarkers that reflects eGFR in a population without CKD.

Previous research has demonstrated that serum AGEs and soluble receptor of AGEs(sRAGE) are independently associated with eGFR in multivariate linear regression analyses, even after adjusting for factors such as sex and inflammatory state (17). Compared to serum-based detection, skin autofluorescence (SAF) has emerged as a preferred method for assessing the tissue accumulation of AGEs due to its cost-effectiveness, non-invasiveness, and convenience of use. Subsequent studies have revealed elevated SAF levels in patients with hypertension, type 1 diabetes (T1D), hemodialysis, peritoneal dialysis and acute renal failure, as well as diabetes mellitus (DM) and CKD groups (18–20). The underlying mechanism for this accumulation may involve the production of reactive oxygen species (ROS) and oxidative stress. Additionally, AGEs can enhance the activity of the renin-angiotensin system and stimulate the production of transforming growth factor-beta (TGF-β)s, contributing to vascular damage and pro-fibrotic responses that ultimately lead to organ damage (10).

Several studies indicate that SAF levels serve as independent risk factors for vascular calcification, cardiovascular events, and all-cause mortality within the CKD population (21, 22). However, SAF detection has recognised limitations; it can be influenced by skin pigmentation, the presence of arteriovenous fistulas, and measurement variations between the two arms (20, 21, 23). Utilizing a scanning confocal biomicroscope to measure lens autofluorescence is a rapid, non-invasive, and reliable technique (18, 24). The procedure is completed in approximately 6 s and requires no fasting or specific physiological preparation by the patient. It is suitable for the majority of patients, provided they do not have significant cataracts or have not undergone lens replacement surgery, as these conditions can interfere with optical readings. The LAF “essay” measures the ratio of green fluorescence to scattered light as the raw qualifications of AGEs (12). While LAF was initially developed to assess diabetes risk, it has been established that lens autofluorescence is elevated in patients with diabetes mellitus, paralleling the accumulation seen in skin autofluorescence (13). Zhang et al. further demonstrated that LAF could identify diabetic kidney disease (DKD) in patients with type 2 diabetes (T2D), finding that LAF levels were negatively associated with eGFR (*p* = 0.019) (14). This study expands the clinical utility of LAF-AGE technology by demonstrating its predictive value for eGFR in a DKD population.

Our findings suggest that lens-based “metabolic memory” may reflect early renal decline even before clinical symptoms or diagnosis manifestations. While LAF was initially developed for diabetes risk, this study expands its clinical utility by demonstrating its predictive value for eGFR in a non-CKD, non-diabetic cohort, establishing its potential as an early-warning indicator for “at-risk” individuals in a general population. In the univariate linear regression analysis, all selected confounders including age, sex, and metabolic markers showed a significant correlation with eGFR (*p* < 0.01). Crucially, the association between LAF-AGEs and eGFR remained significant in the multivariate model (*β* = −43.37, *p* < 0.001), even after adjusting for these confounders. Robust correlations persisted in additional models where LAF-AGEs were adjusted against each individual confounder.

Further analysis revealed a progressive decline in eGFR as age-adjusted AGE levels (AALs) increased. The multivariate analysis for AALs yielded results consistent with the raw LAF-AGE measurements, identifying a significant independent association between higher AALs and lower eGFR (*β* = −1.02, *p* = 0.002). These results reinforce the potential of LAF-based metrics as stable, non-invasive biomarkers for monitoring renal health.

In conclusion, our results demonstrate that LAF-AGEs and AALs are independently associated with eGFR in individuals without clinical CKD or diabetes. This research indicates that non-invasive lens-based assessment could detect early physiological tendencies that precede established kidney disease, offering a new pathway for large-scale preventative screening. Additionally, our analysis identified that female sex, elevated fasting glucose (diabetes), and hyperuricemia (uric acid) are independent factors significantly associated with lower eGFR, consistent with established literature (25, 26). These results contribute to a deeper understanding of the metabolic factors influencing renal health and highlight the utility of lens-based assessments in asymptomatic individuals.

However, several limitations must be acknowledged. First, the cross-sectional nature of this study allows only for the evaluation of associations; therefore, no causal link between AGE accumulation and renal decline can be inferred. Second, while we adjusted for several clinical variables, other potential confounders—such as smoking status, diet, physical activity, cardiovascular history, and socioeconomic factors (e.g., income and education)—were not included in the current analysis and may influence AGE accumulation. Furthermore, optical limitations of the LAF measurement must be considered. The technique is sensitive to cataracts, lens opacities, or prior lens replacement surgery, which can interfere with fluorescence readings and light scattering.

While LAF-AGEs are a promising screening indicator, future research should incorporate ROC curve analysis, sensitivity, and specificity to fully define their diagnostic accuracy for clinical use. To further validate the relationship between lens AGEs and renal decline, longitudinal studies with extended follow-up periods are required. Future research should specifically target populations across CKD stages 3, 4, and 5, as well as patients undergoing hemodialysis or peritoneal dialysis. Investigating the longitudinal variation of LAF-AGEs alongside eGFR decline in these groups is essential to establish LAF-AGEs as a reliable early-warning indicator and predictor of progressive renal dysfunction over time.

## Data Availability

The original contributions presented in the study are included in the article, further inquiries can be directed to the corresponding author.

## References

[1] Tonelli M Wiebe N Culleton B House A Rabbat C Fok M. Chronic kidney disease and mortality risk: a systematic review. J Am Soc Nephrol. (2006) 17(7):2034–47. 10.1681/ASN.2005101085 16738019

[2] Glassock RJ Winearls C. Screening for CKD with eGFR: doubts and dangers. Clin J Am Soc Nephrol. (2008) 3(5):1563–8. 10.2215/CJN.00960208 18667744 PMC4571145

[3] Shlipak MG Matsushita K Arnlov J Inker LA Katz R Polkinghorne KR. Cystatin C versus creatinine in determining risk based on kidney function. N Engl J Med. (2013) 369(10):932–43. 10.1056/NEJMoa1214234 24004120 PMC3993094

[4] Levey AS Stevens LA Schmid CH Zhang YL Castro AF 3rd Feldman HI. A new equation to estimate glomerular filtration rate. Ann Intern Med. (2009) 150(9):604–12. 10.7326/0003-4819-150-9-200905050-00006 19414839 PMC2763564

[5] Steenbeke M De Bruyne S Van Aken E Glorieux G Van Biesen W Himpe J. UV fluorescence-based determination of urinary advanced glycation end products in patients with chronic kidney disease. Diagnostics. (2020) 10(1):34. 10.3390/diagnostics10010034 31936498 PMC7168138

[6] Yamamoto M Sugimoto T. Advanced glycation end products, diabetes, and bone strength. Curr Osteoporos Rep. (2016) 14(6):320–6. 10.1007/s11914-016-0332-1 27704396 PMC5104767

[7] Burd J Lum S Cahn F Ignotz K. Simultaneous noninvasive clinical measurement of lens autofluorescence and rayleigh scattering using a fluorescence biomicroscope. J Diabetes Sci Technol. (2012) 6(6):1251–9. 10.1177/193229681200600603 23294769 PMC3570864

[8] Monnier VM Cerami A. Nonenzymatic browning in vivo: possible process for aging of long-lived proteins. Science. (1981) 211(4481):491–3. 10.1126/science.6779377 6779377

[9] Rabbani N Thornalley PJ. Advanced glycation end products in the pathogenesis of chronic kidney disease. Kidney Int. (2018) 93(4):803–13. 10.1016/j.kint.2017.11.034 29477239

[10] Molinari P Caldiroli L Dozio E Rigolini R Giubbilini P Romanelli MMC. AGEs and sRAGE variations at different timepoints in patients with chronic kidney disease. Antioxidants. (2021) 10(12):1994. 10.3390/antiox10121994 34943097 PMC8698924

[11] Fotheringham AK Gallo LA Borg DJ Forbes JM. Advanced glycation end products (AGEs) and chronic kidney disease: does the modern diet AGE the kidney? Nutrients. (2022) 14(13):2675. 10.3390/nu14132675 35807857 PMC9268915

[12] Yao J Li W Liu Y Wu T Wang D Arezina R. Investigation of the association between lens autofluorescence ratio and diabetes: a cross-sectional study. Photodiagn Photodyn Ther. (2022) 38:102888. 10.1016/j.pdpdt.2022.102888 35490958

[13] Cahn F Burd J Ignotz K Mishra S. Measurement of lens autofluorescence can distinguish subjects with diabetes from those without. J Diabetes Sci Technol. (2014) 8(1):43–9. 10.1177/1932296813516955 24876536 PMC4454118

[14] Zhang XM Gao Y Yang MX Zheng XD Zhang R Wu YY. Exploration of noninvasive detection of advanced glycation end products in the lens to screen for diabetic kidney disease. Front Endocrinol. (2022) 13:892070. 10.3389/fendo.2022.892070 PMC9278348 35846309

[15] O'Callaghan CA Shine B Lasserson DS. Chronic kidney disease: a large-scale population-based study of the effects of introducing the CKD-EPI formula for eGFR reporting. BMJ Open. (2011) 1(2):e000308. 10.1136/bmjopen-2011-000308 PMC3244664 22184586

[16] Fox CS Larson MG Leip EP Culleton B Wilson PW Levy D. Predictors of new-onset kidney disease in a community-based population. J Am Med Assoc. (2004) 291(7):844–50. 10.1001/jama.291.7.844 14970063

[17] Stinghen AE Massy ZA Vlassara H Striker GE Boullier A. Uremic toxicity of advanced glycation end products in CKD. J Am Soc Nephrol. (2016) 27(2):354–70. 10.1681/ASN.2014101047 26311460 PMC4731113

[18] Gryszczynska B Budzyn M Formanowicz D Wanic-Kossowska M Formanowicz P Majewski W. Selected atherosclerosis-related diseases may differentially affect the relationship between plasma advanced glycation End products, receptor sRAGE, and uric acid. J Clin Med. (2020) 9(5):1416. 10.3390/jcm9051416 32397681 PMC7290396

[19] Blanc-Bisson C Velayoudom-Cephise FL Cougnard-Gregoire A Helmer C Rajaobelina K Delcourt C. Skin autofluorescence predicts major adverse cardiovascular events in patients with type 1 diabetes: a 7-year follow-up study. Cardiovasc Diabetol. (2018) 17(1):82. 10.1186/s12933-018-0718-8 29884175 PMC5993997

[20] Wang AY Wong CK Yau YY Wong S Chan IH Lam CW. Skin autofluorescence associates with vascular calcification in chronic kidney disease. Arterioscler Thromb Vasc Biol. (2014) 34(8):1784–90. 10.1161/ATVBAHA.114.303378 24876353

[21] Varikasuvu SR Sulekar H Aloori S Thangappazham B. The association of non-invasive skin autofluorescence measurements with cardiovascular and all-cause mortality in hemodialysis patients: a meta-analysis. Int Urol Nephrol. (2020) 52(9):1757–69. 10.1007/s11255-020-02543-6 32661621

[22] Shardlow A McIntyre NJ Kolhe NV Nellums LB Fluck RJ McIntyre CW. The association of skin autofluorescence with cardiovascular events and all-cause mortality in persons with chronic kidney disease stage 3: a prospective cohort study. PLoS Med. (2020) 17(7):e1003163. 10.1371/journal.pmed.1003163 32658890 PMC7357739

[23] Fraser SD Roderick PJ McIntyre NJ Harris S McIntyre CW Fluck RJ. Skin autofluorescence and all-cause mortality in stage 3 CKD. Clin J Am Soc Nephrol. (2014) 9(8):1361–8. 10.2215/CJN.09510913 24875193 PMC4123399

[24] Pehlivanoglu S Acar N Albayrak S Karakaya M Ofluoglu A. The assessment of autofluorescence of the crystalline lens in diabetic patients and healthy controls: can it be used as a screening test? Clin Ophthalmol. (2018) 12:1163–70. 10.2147/OPTH.S164960 29983542 PMC6027705

[25] Wang L Xu X Zhang M Hu C Zhang X Li C. Prevalence of chronic kidney disease in China: results from the sixth China chronic disease and risk factor surveillance. JAMA Intern Med. (2023) 183(4):298–310. 10.1001/jamainternmed.2022.6817 36804760 PMC9941971

[26] Zhang L Wang F Wang L Wang W Liu B Liu J. Prevalence of chronic kidney disease in China: a cross-sectional survey. Lancet. (2012) 379(9818):815–22. 10.1016/S0140-6736(12)60033-6 22386035

